# Profile of Selected MicroRNAs as Markers of Sex-Specific Anti-S/RBD Response to COVID-19 mRNA Vaccine in Health Care Workers

**DOI:** 10.3390/ijms26157636

**Published:** 2025-08-07

**Authors:** Simona Anticoli, Maria Dorrucci, Elisabetta Iessi, Salvatore Zaffina, Rita Carsetti, Nicoletta Vonesch, Paola Tomao, Anna Ruggieri

**Affiliations:** 1 Reference Center for Gender-Specific Medicine, Istituto Superiore di Sanità [Italian National Institute of Health], 00161 Rome, Italy; simona.anticoli@iss.it (S.A.); elisabetta.iessi@iss.it (E.I.); 2 Department of Infectious Diseases, Istituto Superiore di Sanità [Italian National Institute of Health], 00161 Rome, Italy; maria.dorrucci@iss.it; 3 Occupational Health Unit, Bambino Gesù Children’s Hospital, 00165 Rome, Italy; salvatore.zaffina@opbg.net; 4 Research Area of Immunology, B-Cell Lab, Bambino Gesù Children’s Hospital, 00164 Rome, Italy; rita.carsetti@opbg.net; 5 Department of Occupational and Environmental Medicine, Epidemiology and Hygiene, Italian Workers’ Compensation Authority (INAIL), 00078 Monte Porzio Catone, Rome, Italy; n.vonesch@inail.it (N.V.); p.tomao@inail.it (P.T.)

**Keywords:** health care workers, vaccine, COVID-19, sex, anti-S/RBD, microRNA

## Abstract

Sex-based immunological differences significantly influence the outcome of vaccination, yet the molecular mediators underpinning these differences remain largely elusive. MicroRNAs (miRNAs), key post-transcriptional regulators of gene expression, have emerged as critical modulators of innate and adaptive immune responses. In this study, we investigated the expression profile of selected circulating miRNAs as potential biomarkers of sex-specific humoral responses to the mRNA COVID-19 vaccine in a cohort of health care workers. Plasma samples were collected longitudinally at a defined time point (average 71 days) post-vaccination and analyzed using RT-qPCR to quantify a panel of immune-relevant miRNAs. Anti-spike (anti-S) IgG titers were measured by chemiluminescent immunoassays. Our results revealed sex-dependent differences in miRNA expression dynamics, with miR-221-3p and miR-148a-3p significantly overexpressed in vaccinated female HCWs and miR-155-5p overexpressed in vaccinated males. MiR-148a-3p showed a significant association with anti-S/RBD (RBD: receptor binding domain) IgG levels in a sex-specific manner. Bioinformatic analysis for miRNA targets indicated distinct regulatory networks and pathways involved in innate and adaptive immune responses, potentially underlying the differential immune activation observed between males and females. These findings support the utility of circulating miRNAs as minimally invasive biomarkers for monitoring and predicting sex-specific vaccine-induced immune responses and provide mechanistic insights that may inform tailored vaccination strategies.

## 1. Introduction

The unprecedented global impact of the COVID-19 pandemic has accelerated the development and deployment of vaccines, particularly mRNA-based vaccines, which have proven highly effective in preventing severe disease caused by SARS-CoV-2 [1]. However, emerging evidence suggests that the immune response to these vaccines is not uniform across all individuals, with notable differences observed between males and females [2]. In particular, females generally exhibit higher antibody levels and stronger humoral responses than males [3,4,5].

In recent years, significant progress has been made in detecting biomarkers for vaccine immunogenicity and efficacy [6]. In this context, microRNAs (miRNAs), a class of small, non-coding RNAs involved in the regulation of gene expression at the post-transcriptional level [7], have been shown to be attractive candidates [8,9]. MiRNAsare known to modulate key aspects of the innate and adaptive immune responses, including the activation and differentiation of immune cells, cytokine production, and the regulation of antibody production; they can be secreted and circulate in body fluids, associated with microvesicles, apoptotic bodies, and exosomes, making them stable and easily measurable in blood [10,11]. Given these features, miRNAs are increasingly recognized as potential biomarkers for various diseases and immune responses, including vaccine-induced immunity. Additionally, in humans, around 118 microRNAs are located on the X chromosome (roughly 10% of the total known microRNAs), according to the miRBase database, and only four encoded on the Y chromosome; the X chromosome’s microRNAs are thought to play important roles in various cellular processes, including immunity and cancer [12]. Given the above-mentioned crucial role of microRNAs in regulating important cellular processes and pathways, such as apoptosis, differentiation, oxidative stress, etc., and in immune responses during infection and vaccination, females have a greater regulatory capacity than males.

Despite the growing interest in the role of miRNAs as predictive markers of vaccine response, there is limited understanding of how specific miRNAs might contribute to sex-specific differences in the antibody response to the COVID-19 mRNA vaccine.

This study aims to profile selected miRNAs, known to take part in immune responses to vaccinations based on previous reports [13,14,15,16,17,18], to determine their potential as markers of sex-specific anti-S/RBD (anti-spike/ receptor binding domain) response to the COVID-19 mRNA vaccine. By analyzing miRNA expression in vaccinated individuals, we seek to identify those that associate with differences in anti-S antibody titers between COVID-19 vaccinated male and female individuals.

## 2. Results

### 2.1. Description of the Study Population

The study population was composed of 128 healthcare workers (HCWs), 47 men (36.7%) and 81 women (63.3%), working at the Bambino Gesù Children’s Hospital in Rome, Italy. All participants had no history of SARS-CoV-2 infection and had received two doses of the mRNA vaccine. Overall, the median age was 45 years, slightly higher among female workers (47 years) than male workers (42 years) (Table 1). The majority of HCWs belonged to the oldest age group (46–72 years) for females (70.5%) and the youngest age group (23–45 years) for males (43.3%), according to the age-based classification of the research population.

### 2.2. Sex Differences in Humoral Response to COVID-19 Vaccination

The level of anti-S/RBD antibodies was measured 71 days post-second dose of the COVID-19 vaccine.

As shown in Table 2, vaccinated female HCWs showed significantly higher anti-S/RBD levels (geometric mean 1069 AU/mL [AU: Arbitrary Units], 95% CI: 925.8–1234 AU/mL) than male HCWs (geometric mean 713.9 AU/mL, 95% CI: 509.1–1001 AU/mL). Considering age at vaccination, older male and female HCWs tended to have lower anti-S/RBD antibody (Ab) concentrations (979 AU/mL in females and 526 AU/mL in males) than those of younger subjects (1031 AU/mL in females and 863 AU/mL in males), although the differences observed were not statistically significant (*p* > 0.05).

### 2.3. MiRNA Expression Profile in Plasma from COVID-19 Vaccinated Male and Female HCWs and Their Association with Anti-S/RBD Levels

Six miRNAs (miR-148a-3p, miR-221-3p, miR-150-5p, miR-155-5p, miR-223-3p, and miR-98-5p) were analyzed as potential sex-specific markers of the COVID-19 vaccine response. These miRNAs were selected based on their reported activity in immune responses to vaccinations (all the selected miRNAs), being encoded on the X chromosome (miR-223-3p and miR-98-5p), or having their expression regulated by sex hormones (all the selected miRNAs).

**Figure 1:**
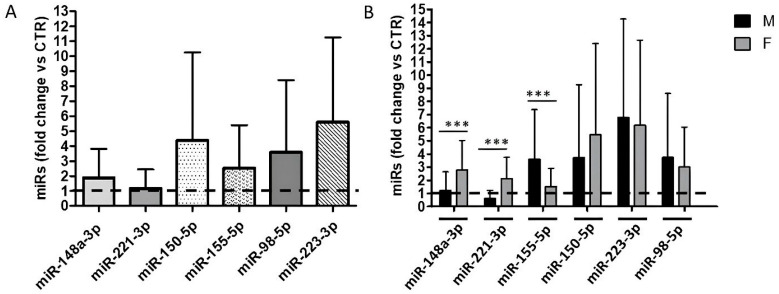
Relative quantitation of the selected microRNAs measured by qRT-PCR. Plasma level of the selected miRNAs in COVID-19 vaccinated HCWs: (**A**) In the overall under-study population, the mean values of fold change for each group are shown relative to the mean CT values of the unvaccinated control population, shown by the dotted horizontal bar; (**B**) in male and female HCWs, the mean values of fold change for each group are shown relative to the mean CT values of the unvaccinated and sex-matched control. The mean ± SD values are shown. Statistically significant differences between sexes were evaluated by the Mann–Whitney U test. *** *p* < 0.001. Obtained with GraphPad Prism (version 5.0).

Significantly higher expression levels of most of the circulating miRNAs analyzed (miR-148a-3p, miR-150-5p, miR-155-5p, miR-98-5p, and miR-223-3p) were detected in the vaccinated total workers cohort compared to the unvaccinated controls, as shown in Figure 1A. The only exception was miR-221-3p, whose expression level seemed to be the same in vaccinated and non-vaccinated HCWs.

However, sex-specific analysis (Figure 1B) revealed that some of the microRNAs had unequal plasma concentrations in male and female vaccinated HCWs. Specifically, miR-148a-3p and miR-221-3p levels increased in female HCWs but remained the same as unvaccinated and sex-matched control subjects in male HCWs after COVID-19 vaccination. Additionally, miR155-5p was significantly overexpressed in vaccinated males, not in females.

**Figure 2:**
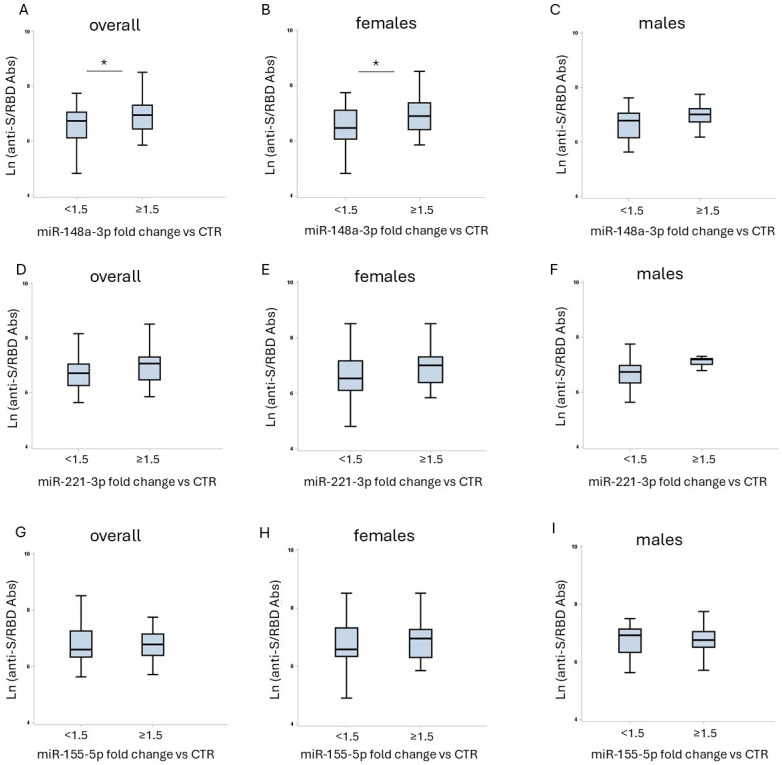
Association between circulating microRNA fold changes and anti-S/RBD antibody levels after vaccination. Box plots show distributions of Ln (anti-S/RBD antibody titer) based on two groups: those with miR-148a-3p fold changes ≥1.5 and <1.5 between vaccinated and unvaccinated total HCWs, (**A**) female (**B**) and male HCWs (**C**); those with miR-221-3p fold changes ≥1.5 and <1.5 between vaccinated and unvaccinated total HCWs (**D**), females (**E**) and males (**F**); those with miR-155-5p fold changes ≥1.5 and <1.5 between vaccinated and unvaccinated total HCWs (**G**), female (**H**) and male HCWs (**I**). The boxes represent the interquartile ranges (IQR): the medians are the lines inside, the first quartile is the bottom edge, and third quartile is the top edge; the lines that extend from each box indicate the range of values that are outside of the IQR and that do not exceed 1.5-fold the IQR. Differences between sexes were tested by *t*-test. * *p* < 0.05.

To investigate the possible association between miRNAs that showed sex-different expression in vaccinated HCWs (miR-148a-3p, miR-221-3p, and miR-155-5p) and anti-S/RBD response following COVID-19 vaccination, we initially grouped subjects with microRNA fold-changes ≥ 1.5, which means an increase in miRNA levels, and <1.5, which means no variation in miRNA expression, comparing vaccinated and unvaccinated HCWs. Anti-S/RBD levels were subsequently compared between the two groups. In the overall vaccinated workers, the group having a fold variation for miR-148a-3p ≥ 1.5 showed significantly higher levels of anti-S/RBD antibodies compared to those in the group with <1.5-fold changes (Figure 2A).

Sex-disaggregated analysis revealed that only female HCWs in the group with miR-148a-3p fold changes ≥ 1.5 showed significantly higher levels of the anti-S/RBD compared to those in the group with <1.5-fold changes (Figure 2B). A similar trend was observed in male workers, but without statistical significance (Figure 2C).

In contrast, neither the entire cohort nor the sex-disaggregated population of healthcare professionals showed a statistically significant difference in anti-S/RBD distributions when the same categorization based on fold variation (≥1.5 and <1.5) was applied for miR-221-3p and miR-155-5p (Figure 2D–I), although they showed a trend similar to that observed with miR-148a-3p.

These results raise the possibility that miR-148a-3p may have a sex-specific role in the anti-S response to COVID-19 immunization.

In Figure S1, the results of a simple regression model are shown, further indicating a strong statistical association between anti-S/RBD antibodies and a ≥ 1.5-fold change in circulating miR-148a-3p. This association was observed following COVID-19 vaccination, but only in female healthcare workers.

### 2.4. Targets of miR-148a-3p, miR-221-3p, and miR-155-5p

To ascertain the biological implications of the variation in miR-148a-3p, miR-221-3p, and miR-155-5p expression associated with sex, we first constructed a network of the miRNA-target interactions that have been experimentally verified, followed by a functional enrichment analysis of the KEGG pathways that comprise the targets (Tables S2–S4 and Table 3, Table 4 and Table 5).

Target genes of miR-148a-3p were discovered to control a total of 75 significantly enriched pathways (FDR < 0.05), as shown in Supplementary Table S1. It is interesting to note that several of these pathways were engaged in modulating the immune response and/or were often involved in response to several viral infections (Table 3). These include, among others, Epstein–Barr virus infection, the FoxO signaling pathway, human T-cell leukemia virus infection, human papillomavirus infection, the TGF-beta signaling pathway, herpes simplex virus 1 infection, measles, apoptosis, hepatitis B, the chemokine signaling pathway, Th17 cell differentiation, and antigen processing and presentation.

miR-221-3p has been reported to promote B-cell proliferation and class switch recombination in vitro by targeting Foxp1 and Arid1a as regulators of Ig class switch recombination (CSR), as well as IgE production in allergic hypersensitivity, which suggested a role for this miRNA as a regulator of cell production of antibodies associated with allergy [19].

Table S3 and Table 4 show the KEGG pathways and miR-221-3p targets involved, indicating that miR-221-3p has a role in the regulation of the immune responses (FoxO signaling, Toll–like receptor signaling, leukocyte migration, Th17 differentiation, and chemokine signaling), sex hormone pathways, cell senescence, and apoptosis, among others.

MiR-155-5p is strongly expressed by hematopoietic cells and acts as a master regulator of immune responses, finely tuning both the innate and adaptive immune systems [20]. Its targets do indeed play a role in controlling inflammatory pathways, responses to viral infections, and Toll-like receptor signaling (TNF signaling pathway, response to HBV, HCV, and EBV viruses, and Toll-like receptor pathways) (Table 5 and Table S4).

**Figure 3:**
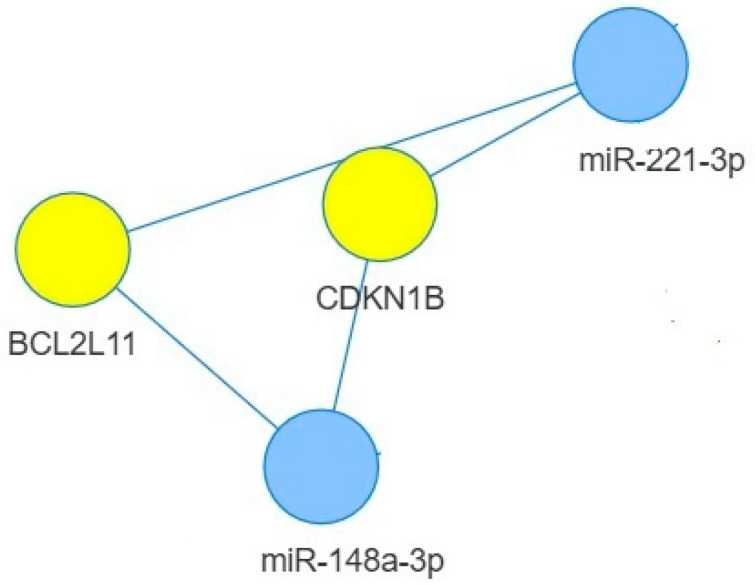
Network analysis and target mRNAs shared between miR-148a-3p and miR-221-3p. The miRNA-target interactions were analyzed by using MIENTURNET. Only targets with “strong interaction” have been considered.

Results presented in Figure 3 highlight experimentally validated targets shared between miR-148a-3p and miR-221-3p, which we found overexpressed in plasma of female HCWs. *CDK1B* and *BCL2L11* emerge as the exclusive, strongly validated common targets between miR-148a-3p and miR-221-3p that are components of the cell cycle and apoptosis pathways.

## 3. Discussion

The present study pointed out a sex-specific response to the two-dose requirement of COVID-19 vaccination in a cohort of healthcare workers. Female individuals showed higher levels of anti-S/RBD antibodies about 71 days after vaccination, compared to males. This finding aligns with our previous research, where we reported a stronger antibody response in females than in males following the COVID-19 mRNA vaccine [5].

Age-related differences in antibody response to vaccination were not observed in either the whole population or sex-disaggregated analysis. This is likely because most of the population was under 45 years old.

The molecular basis of sex disparity in immune responses includes genetic, hormonal, and epigenetic mechanisms. Among the latter, DNA methylation patterns and microRNA regulation of gene expression are reported as potential mechanisms of sex differences in pathophysiology and immune responses. In the present study, we investigated a profile of six microRNAs selected for their role in immune cell regulation and as being encoded on the X chromosome and/or being regulated by sex hormones.

MicroRNAs have been previously investigated as potential markers of vaccine response. The temporal and vaccine-specific expression of microRNAs has been connected to the effectiveness of vaccines or the adverse events linked to vaccination. Atherton et al. considered microRNAs as promising biomarkers that could provide crucial information for vaccine development after identifying microRNA patterns specific to several vaccine types [7]. MicroRNAs found in extracellular vesicles are essential tools for enhancing vaccine efficacy and serving as indicators of immunological response and adverse outcomes after vaccination, according to Oshiumi H. [21]. Furthermore, small regulatory microRNAs crucially regulate cytokines and other significant immune mediators’ production and functions [21,22,23]. Moreover, a correlation between B-cell-specific microRNAs and neutralizing antibody response intensity after measles vaccination has been reported, suggesting that B-cell-specific miRNAs may serve as useful predictive biomarkers of vaccine humoral immune response [17].

Although it is known that microRNAs are expressed differently in males and females, the influence and modification of the immunological response to vaccinations by microRNAs in a sex-specific manner have not been well documented. This sex-biased microRNA expression has been observed in both invertebrates and higher species and is attributed to hormonal and genetic differences between the sexes [24,25]. Specifically, sex steroid hormones, such as estrogens, play a role in regulating miRNA expression [26]. Additionally, there is a difference in the density of miRNAs encoded in sex chromosomes, with the X chromosome containing a significantly higher density of miRNAs than the Y chromosome [12]. MicroRNAs are crucial regulators of gene expression and cell functions, suggesting that females may have more precise regulation of genes involved in immune responses.

In the present study, we were able to identify a sex-specific miRNA signature of response to COVID-19 vaccination with two doses of mRNA vaccine.

We further detected that levels of circulating miR-148a-3p were associated with higher levels of anti-S/RBD response in females more significantly than in males.

miR-148a-3p, encoded on chromosome 7, has been reported to regulate antigen presentation of TLR-triggered dendritic cells and to negatively regulate innate immune response. Considering that dendritic cells are crucial for activation of B-cells through naive T-cell priming, it is conceivable that miR-148a could affect the antibody levels after vaccination [27]. Furthermore, Pracht et al. found that miR-148a-3p promoted differentiation of B-cells to antibody-secreting plasma cells (PC) and modulated PC survival [28]. miR-148a has been reported to be inducible by androgen in prostate cancer cell lines and to be inhibited by estradiol in breast cancer cells, highlighting its sex-specific role in carcinogenesis [29,30].

Here we found that miR-148a-3p, overexpressed in vaccinated female HCWs and positively associated with higher antibody levels in response to vaccination, was involved in the regulation of several pathways identified by the Mienturnet enrichment analysis, including genes of the innate immune responses to several viral infections, of the cell cycle and apoptosis, and of the chemokine and cytokine signaling pathways, as well as antigen presentation and processing, suggesting its multiple regulatory functions.

It should be considered that the same miRNA can have distinct effects depending on the cell type. For miRNA-mediated regulation to be effective, its target genes must be expressed at appropriate levels in the given cell type. If a target gene is not highly expressed in a particular cell type, the miRNA may not have a noticeable effect on it. Thus, the impact of miRNA regulation could be amplified in certain cell types where its targets are more abundant. Therefore, understanding the precise mechanisms by which miR-148a-3p exerts its effect on the humoral response to the COVID-19 vaccine requires a more detailed and in-depth evaluation.

Interestingly, miR-221-3p and miR-155-5p expression levels were also significantly different in male and female HCWs vaccinated with COVID-19 mRNA vaccines. Specifically, miR-221-3p was overexpressed in vaccinated females compared to the unvaccinated and to vaccinated males. miR-155-5p was overexpressed in vaccinated males compared to females. Although we could not detect a statistically significant association between the variation of miR-221-3p and miR-155-5p upon vaccination and antibody levels, it is intriguing that all these microRNAs have a role in immune function. MiR-221-3p has been reported to increase B-cell proliferation and class switch recombination in vitro, suggesting its possible role in regulating the production of antibodies linked to allergies [19]. Furthermore, miR-221-3p has been shown to modulate inflammatory responses in M1 and M2 macrophages activated by TLR4 [31], and its dysregulated expression has been reported to be associated with severe COVID-19 [32]. Specifically, miR-221-3p targets molecules of the inflammatory pathways, such as TLRs, NF-kB, cytokines, and chemokines. Thus, the higher miR-221-3p level in vaccinated females may conceivably affect activation of the innate immune response to vaccine antigens.

MiR-148a-3-p and miR-221-5-p, overexpressed in female plasma, target two common genes, *BCL2L11* and *CDKN1B*, that are involved in apoptosis and cell cycle regulation and are reported to regulate the immune response and antibody production. We can hypothesize that, because of the miRNA upregulation in females, the targets will be downregulated with subsequent activation of B-cell proliferation and antibody production more actively in female than in male vaccinees.

MiR-155-5p is one of the most studied microRNAs. It is highly expressed in hematopoietic cells, where it plays a key role in inflammation and immunity [20,33]. Its targets are involved in the regulation of inflammatory pathways, the response to viral infections, and Toll-like receptor (TLR) signaling. It acts as a key regulator of both innate and adaptive immunity, influencing various immune cell types, including T-cells, B-cells, macrophages, and dendritic cells. Since miR-155-5p is overexpressed in vaccinated male individuals compared to females, it is conceivable to hypothesize that it could be a male-specific immune modulator in response to COVID-19 vaccination. However, this point requires further and targeted investigation.

So far, a limited number of studies have documented variations in microRNAs after vaccination [13,14,15,16,17,18]. In line with our results, Miyashita et al. [13] were able to identify that miR-148a was involved in antibody production in response to the COVID-19 mRNA vaccine, and miR-92a-2-5p was related to cytokines and adverse reactions to vaccination. An association between miRNA expression and local adverse reactions to influenza vaccination has also been reported [22], with levels of serum vesicle miR-451a negatively correlated to inflammatory cytokines after seasonal influenza vaccination. The aforementioned studies analyzed microRNAs before vaccination, making them predictive of the response to vaccination. In the present study, we analyzed miRNA levels in the plasma 71 days post-second dose of the COVID-19 vaccine, providing an observational report on microRNA expression associated with the vaccine response. This can be valuable as it provides evidence of miRNAs associated with antibody production in response to vaccination, but it is also limited by the lack of comparative evaluation before and after vaccination. Another limitation of this study is the small sample size, particularly as we disaggregated the analysis by sex. Nevertheless, we consider the analysis of data by sex to be a strength and original aspect of the study compared to previously published reports.

## 4. Materials and Methods

### 4.1. Population and Study Design

In this prospective, observational study, we recruited 128 healthcare workers (HCWs) working at the Bambino Gesù Children’s Hospital in Rome who received the two-dose schedule of the BNT162b COVID-19 vaccine in January–March 2021 and have never had a SARS-CoV-2 infection.

Plasma samples were collected from HCWs recruited between 50 and 100 days (median 71 days) after receiving their second dose of the COVID-19 vaccine. This was performed concurrently with periodic health surveillance. The samples were then used to measure plasma levels of antibodies against the SARS-CoV-2 spike/RBD antigen (anti-S/RBD) using a commercial serologic assay (AdviseDx SARS-CoV-2 immunoglobulin (Ig)G II assay, ARCHITECT^®^, i2000sr Abbott Diagnostics, Abbott, IL, USA) following the manufacturers’ instructions.

Demographic characteristics of the enrolled subjects are reported in Table 1.

This study was conducted after obtaining ethical approval from ISS (AOO-ISS 09/05/2021–0017778).

Before enrollment, all study participants provided written informed consent.

### 4.2. RNA Extraction, cDNA Synthesis, and Quantitative Analysis by qRT-PCR of the Selected MicroRNAs

The level of selected circulating miRNAs was evaluated in plasma samples from a subgroup of 86 HCWs (43 males, 43 females) whose demographic characteristics are reported in Table S1.

Total RNA from plasma samples was extracted using a “Plasma/Serum Isolation kit” (Norgen Biotek, Thorold, ON, Canada), according to the manufacturer’s instructions. miRNAs were reverse transcribed by the “TaqMan™ Advanced miRNA cDNA Synthesis Kit” (Thermo Fisher^TM^, Waltham, MA, USA). Quantitative expression of the selected microRNAs was carried out by Real-Time Quantitative Polymerase Chain Reaction (RT-qPCR) using specific inventoried Advanced TaqMan MicroRNA Assays (Thermo Fisher^TM^, Waltham, MA, USA). The reactions were run in a Quant Studio 12K Flex (Thermo Fisher^TM^, Waltham, MA, USA) qPCR Real-Time PCR machine. Fold expression changes of miRNAs, relative to sex-matched controls who did not receive the COVID-19 vaccine, were determined by the 2^−ΔΔCt^ method, after normalization to the spike-in cel-miR-39. A 1.5-fold increase in miRNA level or a 0.5-fold decrease was considered a significant variation in the relative quantitation of microRNAs between vaccinated and unvaccinated subjects.

### 4.3. Statistical Analyses

Anti-S/RBD concentrations (AU/mL) were reported as geometric means with their confidence intervals (95% CI).

When the variables of interest were not normally distributed, analyses were performed after a natural logarithmic transformation, and when comparing groups, we applied t-tests, whilst we performed Mann–Whitney non-parametric tests for original data. A *p*-value of <0.05 was considered statistically significant.

In particular, for each miRNA, we created a categorical variable choosing the value of 1.5-fold change (FC) between vaccinated and unvaccinated controls, which is considered the biological significance threshold, as a cut-off.

To study the effect of miRNAs on anti-S/RBD antibodies, we applied a simple regression model considering the natural logarithm (ln) of anti-S/RBD antibody level as the response variable and miRNA fold change as a categorical covariate; we showed LS-means (least-square means) of the response variable by miR cutoff, overall and by sex. In particular, we applied the Laplace approximation to fit the regression models.

Statistical analysis was performed with SAS version 9.4 and GraphPad Prism (version 5.0).

### 4.4. MicroRNA-Target Interaction Network

The miRNA-target interactions were analyzed by using MIENTURNET (MicroRNA ENrichment TURned NETwork; http://userver.bio.uniroma1.it/apps/mienturnet/, accessed on 1 June 2025) [34], a web application that enables users to apply filters based on evidence categories from the miRTarBase database, distinguishing between ‘Strong’ experimental methods (e.g., Luciferase assay, Western), ‘Weak’ evidence (e.g., CLIP), or a combination of both (‘Strong and Weak’). In this work, we have used the ‘Strong’ experimental method and set the threshold for the false discovery rate (FDR) as 0.05.

### 4.5. Functional Enrichment Analysis

The functional enrichment analysis was performed by querying the Kyoto Encyclopedia of Genes and Genomes (KEGG) pathway through the MIENTURNET web tool; *p*-values were adjusted with the Benjamini–Hochberg method, and a threshold (FDR) equal to 0.05 was set to identify functional annotations significantly enriched among genes of the input list.

## 5. Conclusions

In conclusion, our study offers an original contribution to understanding microRNAs associated with the immune response to COVID-19 vaccination, highlighting the potential role of specific microRNAs in antibody production. Understanding these associations could not only enhance our knowledge of the immune response to COVID-19 vaccines but also contribute to paving the way for more personalized, sex-specific approaches to vaccination, ultimately enhancing the effectiveness of vaccines in diverse populations and possibly reducing the adverse events of vaccinations. Although limited by a small sample size and the absence of pre-vaccination data, the post-vaccination analysis and sex-stratified approach, which complement previous predictive studies, represent innovative and valuable aspects of the work. Future longitudinal studies with larger cohorts will be essential to confirm and extend these findings.

## Figures and Tables

**Table 1:** Demographic characteristics of the study population.

	Study Population	Males	Females
	*n* = 128	47 (36.7%)	81 (63.3%)
Age (years), median (IQR); (range)	45 (36–54); (23–72)	42 (36–51); (26–72)	47 (35.5–54.5); (23–64)
**Age Groups**			
23–45 years (*n*)	67 (52.3%)	29 (43.3%)	38 (56.7%)
46–72 years (*n*)	61 (47.7%)	18 (29.5%)	43 (70.5%)
Interval (days) median (IQR); (range)	71 (70–79); (55–100)	71 (70–81); (55–100)	71 (69–78); (56–100)

IQR: interquartile range; interval: time interval between the second vaccine dose and anti-S/RBD testing.

**Table 2:** Anti-S/RBD concentrations by sex and age. Geometric means (GMT) of anti-S/RBD levels (AU/mL) after the second dose of COVID-19 vaccine (median time interval: 71 days); *p*-values refer to the comparison among groups.

	Anti-S/RBD Titer (AU/L) GMT (95% CI)	*p*-Value
**All subjects**	921.5	
	(790.7–1074)	
**F**	1069 (925.8–1234)	0.0123
**M**	713.9	
	(509.1–1001)	
**Age**		
**≤45 years**	1031	0.42
	(925.8–1234)	
**>45 years**	713.9	
	(509.1–1001)	
**Sex and age**		
**F ≤ 45 years**	1031	0.37
	870.7–1220	
**F > 45 years**	978.9	
	830.9–1153	
**M ≤ 45 years**	862.7	0.48
	(696.5–1068)	
**M > 45 years**	526.2	
	(225.6–1227)	

**Table 3:** KEGG pathways enrichment analysis for miR-148a-3p. Statistically significant pathways (FDR ≤ 0.05) involved in immune response modulation and/or frequently altered in response to viral infections.

Description	KEGG ID	*p*-Value	FDR *	Genes
**Epstein–Barr virus infection**	hsa05169	8.74364 × 10^−8^	2.49 × 10^−6^	*HLA-G*/*BCL2*/*CDKN1B*/*RUNX3*/ *BCL2L11*/*PDIA3*/*STAT3*/*IKBKB*/*BAX*
**FoxO signaling pathway**	hsa04068	8.86186 × 10^−7^	1.37 × 10^−5^	*IRS1*/*CDKN1B*/*BCL2L11*/*S1PR1*/*STAT3*/ *TGFB2*/*IKBKB*
**Human T-cell leukemia virus 1 infection**	hsa05166	2.79378 × 10^−5^	0.000259	*HLA-G*/*MMP7*/*SMAD2*/*TGFB2*/*IKBKB*/ *BAX*/*NRP1*
**Human papillomavirus infection**	hsa05165	4.59073 × 10^−5^	0.000387	*HLA-G*/*WNT10B*/*CDKN1B*/*ITGB8*/*ITGA5*/ *WNT1*/*IKBKB*/*BAX*
**TGF-beta signaling pathway**	hsa04350	7.06962 × 10^−5^	0.000504	*TGIF2*/*ACVR1*/*ROCK1*/*SMAD2*/*TGFB2*
**PI3K-Akt signaling pathway**	hsa04151	8.29241 × 10^−5^	0.000517	*IRS1*/*BCL2*/*CDKN1B*/*ITGB8*/*ITGA5*/*MET*/ *BCL2L11*/*IKBKB*
**Herpes simplex virus 1 infection**	hsa05168	8.36848 × 10^−5^	0.000517	*HLA-G*/*BCL2*/*ITGA5*/*PDIA3*/*IKBKB*/*BAX*
**MAPK signaling pathway**	hsa04010	0.000177249	0.000912	*RPS6KA5*/*CDC25B*/*MAP3K4*/*MET*/*MAP3K9*/ *TGFB2*/*IKBKB*
**Measles**	hsa05162	0.000232811	0.001135	*BCL2*/*CDKN1B*/*STAT3*/*IKBKB*/*BAX*
**Human cytomegalovirus infection**	hsa05163	0.000274049	0.001156	*HLA-G*/*ROCK1*/*PDIA3*/*STAT3*/*IKBKB*/*BAX*
**Apoptosis—multiple species**	hsa04215	0.000288247	0.001161	*BCL2*/*BCL2L11*/*BAX*
**Hepatitis B**	hsa05161	0.000486342	0.001733	*BCL2*/*STAT3*/*TGFB2*/*IKBKB*/*BAX*
**Apoptosis**	hsa04210	0.00219604	0.005812	*BCL2*/*BCL2L11*/*IKBKB*/*BAX*
**Adipocytokine signaling pathway**	hsa04920	0.002859899	0.006971	*IRS1*/*STAT3*/*IKBKB*
**p53 signaling pathway**	hsa04115	0.003478888	0.007952	*BCL2*/*SERPINE1*/*BAX*
**Cellular senescence**	hsa04218	0.003692136	0.007952	*HLA-G*/*SERPINE1*/*SMAD2*/*TGFB2*
**Cell cycle**	hsa04110	0.003777087	0.007952	*CDC25B*/*CDKN1B*/*SMAD2*/*TGFB2*
**mTOR signaling pathway**	hsa04150	0.003777087	0.007952	*IRS1*/*WNT10B*/*WNT1*/*IKBKB*
**Chemokine signaling pathway**	hsa04062	0.00765295	0.014769	*VAV2*/*ROCK1*/*STAT3*/*IKBKB*
**Th17 cell differentiation**	hsa04659	0.009837214	0.017193	*SMAD2*/*STAT3*/*IKBKB*
**Hormone signaling**	hsa04081	0.011813777	0.020265	*CCKBR*/*IRS1*/*ACVR1*/*STAT3*
**Regulation of actin cytoskeleton**	hsa04810	0.014353241	0.024174	*ITGB8*/*VAV2*/*ITGA5*/*ROCK1*
**Antigen processing and presentation**	hsa04612	0.043197093	0.053352	*HLA-G*/*PDIA3*

* FDR: false discovery rate.

**Table 4:** KEGG pathways enrichment analysis for miR-221-3p targets. Statistically significant pathways (FDR ≤ 0.05) involved in immune response modulation and/or hormone signaling pathways.

Description	KEGG ID	*p*-Value	FDR *	Genes
**FoxO signaling pathway**	hsa04068	1.02 × 10^−7^	4.28 × 10^−6^	*CDKN1B*/*BCL2L11*/*FOXO3*/*TNFSF10*/*BNIP3*/ *PTEN*/*SIRT1*/*MDM2*/*PIK3R1*
**Cellular senescence**	hsa04218	4.82 × 10^−6^	4.84 × 10^−5^	*FOXO3*/*PTEN*/*TP53*/*ETS1*/*RB1*/*SIRT1*/*MDM2*/*PIK3R1*
**Apoptosis**	hsa04210	1.87 × 10^−5^	0.000141	*BCL2L11*/*BBC3*/*TNFSF10*/*FOS*/*TP53*/*APAF1*/*PIK3R1*
**Mitophagy—animal**	hsa04137	4.31 × 10^−5^	0.000217	*FOXO3*/*BNIP3L*/*TBK1*/*BNIP3*/*TP53*/*BECN1*
**Autophagy—animal**	hsa04140	7.54 × 10^−5^	0.000325	*DDIT4*/*TBK1*/*BNIP3*/*PTEN*/*RAB1A*/*PIK3R1*/*BECN1*
**p53 signaling pathway**	hsa04115	9.46 × 10^−5^	0.000329	*BBC3*/*PTEN*/*TP53*/*APAF1*/*MDM2*
**PI3K-Akt signaling pathway**	hsa04151	0.00034	0.000906	*CDKN1B*/*BCL2L11*/*FOXO3*/*KIT*/*DDIT4*/*PTEN*/ *TP53*/*MDM2*/*PIK3R1*
**Cell cycle**	hsa04110	0.000409	0.00103	*CDKN1B*/*CDKN1C*/*TP53*/*WEE1*/*RB1*/*MDM2*
**TNF signaling pathway**	hsa04668	0.000815	0.001891	*ICAM1*/*FOS*/*SELE*/*PIK3R1*/*SOCS3*
**mTOR signaling pathway**	hsa04150	0.002861	0.004887	*DDIT4*/*PTEN*/*DVL2*/*PIK3R1*/*GRB10*
**Toll-like receptor signaling pathway**	hsa04620	0.004558	0.007239	*TBK1*/*FOS*/*TICAM1*/*PIK3R1*
**Leukocyte transendothelial migration**	hsa04670	0.005682	0.008869	*ICAM1*/*PIK3R1*/*MMP2*/*CXCL12*
**Thyroid hormone signaling pathway**	hsa04919	0.006783	0.010066	*ESR1*/*TP53*/*MDM2*/*PIK3R1*
**Estrogen signaling pathway**	hsa04915	0.010646	0.014602	*FOS*/*ESR1*/*PIK3R1*/*MMP2*
**JAK-STAT signaling pathway**	hsa04630	0.020074	0.025239	*PIK3R1*/*STAT5A*/*SOCS3*/*SOCS1*
**NF-kappa B signaling pathway**	hsa04064	0.027183	0.031548	*ICAM1*/*TICAM1*/*CXCL12*
**Th17 cell differentiation**	hsa04659	0.029922	0.033859	*FOS*/*RUNX1*/*STAT5A*
**Chemokine signaling pathway**	hsa04062	0.031384	0.034647	*FOXO3*/*PAK1*/*PIK3R1*/*CXCL12*
**MAPK signaling** **pathway**	hsa04010	0.037664	0.040358	*KIT*/*FOS*/*TP53*/*PAK1*/*STMN1*
**AMPK signaling** **pathway**	hsa04152	0.039796	0.040938	*FOXO3*/*SIRT1*/*PIK3R1*
**T-cell receptor signaling pathway**	hsa04660	0.039796	0.040938	*FOS*/*PAK1*/*PIK3R1*

* FDR: false discovery rate

**Table 5:** KEGG pathways enrichment for miR-155-5p. Statistically significant pathways (FDR ≤ 0.05) involved in immune response modulation and/or hormone signaling pathways and/or frequently altered in response to viral infections.

Description	KEGG ID	*p*-Value	FDR *	Genes
**Hepatitis B**	hsa05161	6.35 × 10^−13^	2.25 × 10^−11^	*TAB2*/*IKBKE*/*KRAS*/*JUN*/*FADD*/*MYD88*/*YWHAZ*/ *SMAD4*/*APAF1*/*SMAD3*/*CXCL8*/*NFKB1*/*E2F2*/*PIK3R1*/*FOS*/*MAPK14*/*MYC*/*MAPK13*/*STAT1*/ *CASP3*
**Epstein–Barr virus infection**	hsa05169	4.26 × 10^−11^	1.13 × 10^−9^	*TAB2*/*IKBKE*/*ICAM1*/*JUN*/*FADD*/*MYD88*/*RAC1*/ *SAP30L*/*MAP3K14*/*APAF1*/*CCND1*/*NFKB1*/*E2F2*/*PIK3R1*/*MAPK14*/*MYC*/*MAPK13*/*STAT1*/ *CCND2*/*CASP3*
**Cellular senescence**	hsa04218	2.48 × 10^−9^	2.93 × 10^−8^	*RHEB*/*FOXO3*/*KRAS*/*ETS1*/*SMAD2*/*SMAD3*/*CCND1*/*CXCL8*/*NFKB1*/*E2F2*/*PIK3R1*/*MAPK14*/*MYC*/*MAPK13*/*CCND2*/*PTEN*
**TNF signaling pathway**	hsa04668	4.03 × 10^−9^	3.89 × 10^−8^	*TAB2*/*CEBPB*/*EDN1*/*ICAM1*/*SELE*/*JUN*/*FADD*/*MAP3K14*/ *NFKB1*/*PIK3R1*/*FOS*/*MAPK14*/*MAPK13*/*CASP3*
**Toll-like receptor signaling pathway**	hsa04620	1.26 × 10^−8^	9.28 × 10^−8^	*TAB2*/*IKBKE*/*JUN*/*FADD*/*MYD88*/*RAC1*/*CXCL8*/*NFKB1*/*PIK3R1*/*FOS*/*MAPK14*/*MAPK13*/*STAT1*
**IL-17 signaling pathway**	hsa04657	2.44 × 10^−8^	1.62 × 10^−7^	*TAB2*/*CEBPB*/*IKBKE*/*JUN*/*FADD*/*IL17RB*/*CXCL8*/*NFKB1*/ *FOS*/*MAPK14*/*MAPK13*/*CASP3*
**Hepatitis C**	hsa05160	1.68 × 10^−7^	9.4 × 10^−7^	*CLDN1*/*IKBKE*/*KRAS*/*FADD*/*YWHAZ*/*APAF1*/*CCND1*/ *NFKB1*/*E2F2*/*PIK3R1*/*MYC*/*STAT1*/*CASP3*/ *NR1H3*
**Measles**	hsa05162	2.35 × 10^−7^	1.25 × 10^−6^	*TAB2*/*IKBKE*/*JUN*/*FADD*/*MYD88*/*APAF1*/*CCND1*/*NFKB1*/*PIK3R1*/*FOS*/*STAT1*/*CCND2*/*CASP3*
**Th17 cell differentiation**	hsa04659	9.74 × 10^−7^	4.14 × 10^−6^	*IFNGR1*/*SMAD2*/*JUN*/*SMAD4*/*SMAD3*/*NFKB1*/ *FOS*/*MAPK14*/*HIF1A*/*MAPK13*/*STAT1*
**FoxO signaling pathway**	hsa04068	1.03 × 10^−6^	4.23 × 10^−6^	*FOXO3*/*KRAS*/*BCL6*/*SMAD4*/*GABARAPL1*/*SMAD3*/ *CCND1*/*PIK3R1*/*MAPK14*/*MAPK13*/*CCND2*/ *PTEN*
**T-cell receptor** **signaling pathway**	hsa04660	3 × 10^−6^	1.1 × 10^−5^	*RHOA*/*KRAS*/*JUN*/*CARD11*/*MAP3K14*/*PAK2*/*NFKB1*/*PIK3R1*/*FOS*/*MAPK14*/*MAPK13*
**MAPK signaling pathway**	hsa04010	4.1 × 10^−6^	1.28 × 10^−5^	*TAB2*/*FGF7*/*KRAS*/*CSF1R*/*JUN*/*MAP3K10*/*MYD88*/*RAC1*/*RAPGEF2*/*MAP3K14*/*PAK2*/*NFKB1*/*FOS*/*MAPK14*/*MYC*/*MAPK13*/*CASP3*
**NOD-like receptor signaling pathway**	hsa04621	7.68 × 10^−6^	2.27 × 10^−5^	*TAB2*/*PKN2*/*RHOA*/*IKBKE*/*JUN*/*FADD*/*MYD88*/*GABARAPL1*/*CXCL8*/*NFKB1*/*MAPK14*/*MAPK13*/*STAT1*
**Cell cycle**	hsa04110	3.56 × 10^−5^	8.22 × 10^−5^	*TRIP13*/*SMAD2*/*ANAPC16*/*YWHAZ*/*SMAD4*/ *SMAD3*/*WEE1*/*CCND1*/*E2F2*/*MYC*/*CCND2*
**PI3K-Akt signaling pathway**	hsa04151	4.75 × 10^−5^	0.000108	*RHEB*/*PKN2*/*FGF7*/*FOXO3*/*KRAS*/*MYB*/*CSF1R*/ *NOS3*/*YWHAZ*/*RAC1*/*CCND1*/*NFKB1*/*PIK3R1*/ *MYC*/*RPTOR*/*CCND2*/*PTEN*
**Influenza A**	hsa05164	8.15 × 10^−5^	0.00017	*IKBKE*/*IFNGR1*/*ICAM1*/*FADD*/*MYD88*/*APAF1*/ *CXCL8*/*NFKB1*/*PIK3R1*/*STAT1*/*CASP3*
**B-cell receptor** **signaling pathway**	hsa04662	8.48 × 10^−5^	0.000172	*INPP5D*/*KRAS*/*JUN*/*CARD11*/*RAC1*/*NFKB1*/ *PIK3R1*/*FOS*
**Apoptosis**	hsa04210	0.000274	0.000448	*KRAS*/*JUN*/*FADD*/*MAP3K14*/*APAF1*/*NFKB1*/ *PIK3R1*/*FOS*/*CASP3*
**TGF-beta signaling pathway**	hsa04350	0.000281	0.000453	*SMAD5*/*SMAD1*/*RHOA*/*SMAD2*/*SKI*/*SMAD4*/ *SMAD3*/*MYC*
**Coronavirus disease—COVID-19**	hsa05171	0.000346	0.000536	*TAB2*/*AGTR1*/*IKBKE*/*JUN*/*MYD88*/*CXCL8*/*NFKB1*/ *PIK3R1*/*FOS*/*MAPK14*/*MAPK13*/*STAT1*
**Th1 and Th2 cell differentiation**	hsa04658	0.000617	0.000887	*IFNGR1*/*JUN*/*NFKB1*/*FOS*/*MAPK14*/*MAPK13*/ *STAT1*
**RIG-I-like receptor signaling pathway**	hsa04622	0.000892	0.001193	*IKBKE*/*FADD*/*CXCL8*/*NFKB1*/*MAPK14*/*MAPK13*
**JAK-STAT** **signaling pathway**	hsa04630	0.001268	0.001607	*SOCS1*/*IFNGR1*/*IL13RA1*/*CCND1*/*PIK3R1*/*MYC*/ *SOCS6*/*STAT1*/*CCND2*
**NF-kappa B** **signaling pathway**	hsa04064	0.00127	0.001607	*TAB2*/*ICAM1*/*MYD88*/*CARD11*/*MAP3K14*/ *CXCL8*/*NFKB1*
**Mitophagy—animal**	hsa04137	0.00127	0.001607	*FOXO3*/*KRAS*/*JUN*/*MITF*/*GABARAPL1*/*TOMM20*/*HIF1A*
**Human papillomavirus infection**	hsa05165	0.002137	0.002497	*APC*/*RHEB*/*IKBKE*/*KRAS*/*CSNK1A1*/*FADD*/ *CCND1*/*NFKB1*/*PIK3R1*/*STAT1*/*CCND2*/*CASP3*/*PTEN*
**Leukocyte** **transendothelial migration**	hsa04670	0.002257	0.002608	*CLDN1*/*RHOA*/*ICAM1*/*RAC1*/*PIK3R1*/*MAPK14*/*MAPK13*
**Autophagy—animal**	hsa04140	0.005021	0.005561	*RHEB*/*KRAS*/*GABARAPL1*/*VPS18*/*PIK3R1*/*HIF1A*/*RPTOR*/*PTEN*
**p53 signaling** **pathway**	hsa04115	0.006329	0.006662	*APAF1*/*CCND1*/*CCND2*/*CASP3*/*PTEN*
**Chemokine** **signaling pathway**	hsa04062	0.010868	0.010727	*RHOA*/*FOXO3*/*KRAS*/*RAC1*/*CXCL8*/*NFKB1*/*PIK3R1* /*STAT1*
**Apoptosis—multiple species**	hsa04215	0.013437	0.012642	*FADD*/*APAF1*/*CASP3*
**Natural killer cell mediated** **cytotoxicity**	hsa04650	0.018674	0.016969	*IFNGR1*/*KRAS*/*ICAM1*/*RAC1*/*PIK3R1*/*CASP3*
**Hormone signaling**	hsa04081	0.056541	0.047333	*SMAD5*/*SMAD1*/*RHOA*/*AGTR1*/*SMAD4*/*PIK3R1*/*THRB*

* FDR: false discovery rate.

## Data Availability

The data presented in this study are available upon request from the corresponding author.

## Supplementary Materials

The following supporting information can be downloaded at https://www.mdpi.com/article/10.3390/ijms26157636/s1.
