# Supplementary material for: Profile of Selected MicroRNAs as Markers of Sex-Specific Anti-S/RBD Response to COVID-19 mRNA Vaccine in Health Care Workers
Source: Int J Mol Sci. 2025 Aug 7;26(15):7636. doi: 10.3390/ijms26157636 (PMC12346932; doi:10.3390/ijms26157636)
Supplement: Supplementary file 1 [file ijms-26-07636-s001.zip › Figure S1.pdf]

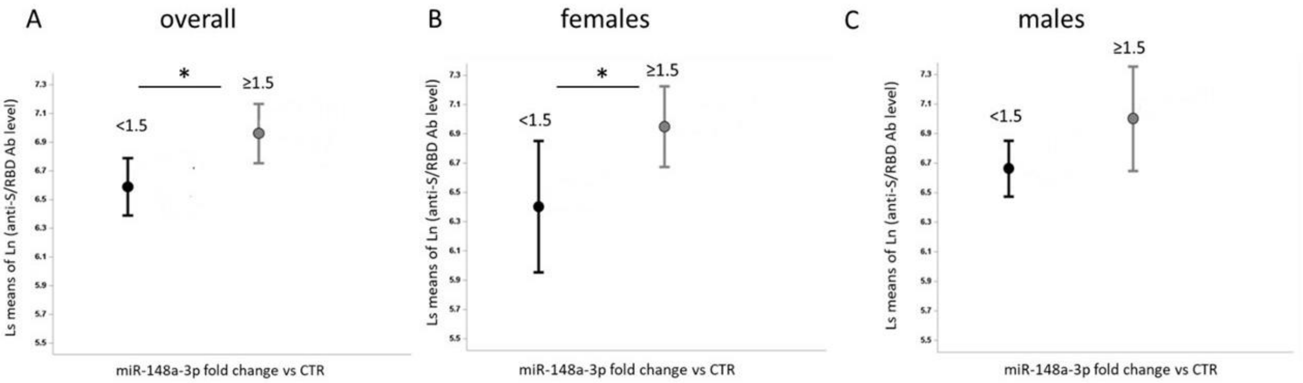

Figure S1. Association between miR-148a-3p fold change and anti-S/RBD level. Simple regression models were applied using ln (anti-S/RBD antibody) levels as the response variable and miR-148a-3p (≥1.5 vs. <1.5 fold change versus unvaccinated control) as the covariate; LS-means (least-squares means) with its 95% CI are shown in each panel: A) overall study population; test for difference ± 0.370, *p*-value = 0.014; B) female HCWs; test for difference ± 0.547, *p*-value = 0.044; C) male HCWs; test for difference ± 0.337, *p*-value = 0.102. *P*-values were calculated by the Mann–Whitney U test. * $p < 0.05$.
