# Supplementary material for: Profile of Selected MicroRNAs as Markers of Sex-Specific Anti-S/RBD Response to COVID-19 mRNA Vaccine in Health Care Workers
Source: Int J Mol Sci. 2025 Aug 7;26(15):7636. doi: 10.3390/ijms26157636 (PMC12346932; doi:10.3390/ijms26157636)
Supplement: Supplementary file 1 [file ijms-26-07636-s001.zip › Table S1.pdf]

Table S1. Demographic characteristics of the 86 vaccinated HCWs and the 40 unvaccinated whose plasma was used for circulating miRNAs analysis

	**All subjects**	**Males**	**Females**
	**n = 86**	**n=43**	**n=.43**
Age (years), median (IQR); (range)	41.5 (34-54); (23-72)	42 (36-51); (26-72)	38 (32-54); (23-62)
**Age groups**	**n (%)**	**n (%)**	**n (%)**
23–45 years	50 (58.1%)	27 (62.8%)	23 (53.5%)
46–72 years	36 (41.9%)	16 (37.2 %)	20 (46.5%)
Interval (days) median (IQR); (range)	71 (69-78); (64-100)	71 (69-78); (64-100)	71 (70-78); (68-85)
**Unvaccinated HCWs (used as controls , CTR)**			
	**n=40**	**n=20**	**n=20**
Age (years), median (IQR); (range)	45 (33-52.2); (26-64)	42 (35-58); (28-64)	45 (30-51); (26-59)
**Age groups**	**n**	**n**	**n**
23–45 years	20	11	9
46–64 years	20	9	11

IQR: interquartile range; Interval: time interval between the second vaccine dose and anti-S/RBD testing.
